# IUC24392-85 Real-world use of erdafitinib in urothelial carcinoma: an Italian multicenter retrospective study

**DOI:** 10.1093/oncolo/oyaf248.024

**Published:** 2025-08-29

**Authors:** A Dri, A Polymeropoulos, E Lai, F Pierantoni, M Ghidini, A Mennitto, A Zivi, E Tassinari, R Cengarle, L Fratino

**Affiliations:** Department of Medical Oncology, Centro di Riferimento Oncologico di Aviano CRO, IRCCS, Aviano, Italy; Biostatistics for clinical research unit, Fondazione IRCCS Istituto Nazionale dei Tumori, Milan, Italy; Oncology Unit 3, Department of Oncology, Istituto Oncologico Veneto IOV—IRCCS, Padua, Italy; Oncology Unit 3, Department of Oncology, Istituto Oncologico Veneto IOV—IRCCS, Padua, Italy; Medical Oncology, Fondazione IRCCS Ca’ Granda, Ospedale Maggiore Policlinico, Milan, Italy; Department of Medical Oncology, Azienda Ospedaliera Universitaria “Maggiore Della Carità”, Novara, Italy; Section of Innovation Biomedicine-Oncology Area, Department of Engineering for Innovation Medicine DIMI, University of Verona and University and Hospital Trust AOUI of Verona, Italy; Medical Oncology, IRCCS Azienda Ospedaliero-Universitaria di Bologna, Bologna, Italy; Department of Medical and Surgical Sciences, University of Bologna, Bologna, Italy; Oncology Unit, ASST di Mantova, Presidio di Mantova, Mantova, Italy; Department of Medical Oncology, Centro di Riferimento Oncologico di Aviano CRO, IRCCS, Aviano, Italy

## Abstract

**Background:**

The study aims to generate real-world evidence on treatment patterns, outcomes, and adverse events (AEs) related to erdafitinib, a pan-fibroblast growth factor receptor (FGFR) inhibitor, in routine clinical practice. Erdafitinib demonstrated superior efficacy over standard chemotherapy in previously treated patients with FGFR2/3-altered metastatic urothelial carcinoma (mUC), but data are limited outside clinical trials.

**Methods:**

This is a retrospective, multicenter, observational study conducted across 13 Italian centers. Survival outcomes were estimated using the Kaplan–Meier method.

**Results:**

Thirty-one patients treated with erdafitinib between 2017 and 2025 were analyzed. Median age was 67 years (IQR 62.5-73). The primary site was the bladder in 18 (58.1%) patients. Twenty (64.5%) patients had de novo mUC. Thirty (98.6%) and 26 (83.9%) patients received prior platinum-based chemotherapy and an immune checkpoint inhibitor, achieving an overall response rate (ORR) in 51.6% and 12.9% of cases, respectively. Hyperphosphatemia occurred in 21 (67.7%) patients, ocular AEs in 15 (53.6%), and nail-related AEs in 16 (53.3%). Median progression-free survival (mPFS) was 6.4 months (95% CI, 4.4-13.8) and median overall survival (mOS) was 12.5 months (95% CI, 8.5-NR), with a median follow-up of 24.1 months (95% CI, 8.7-NR). Patients experiencing AEs had improved mPFS and mOS (Table 1). Of 22 patients who progressed to erdafitinib, 5 received subsequent enfortumab vedotin (EV), while one received EV before erdafitinib. At progression to erdafitinib, mOS was 25.7 months (95% CI, 10.4-NR) with EV and 25 months (95% CI, 8.5-NR) with other treatment.

**Conclusions:**

survival outcomes with erdafitinib are at least comparable to those of EV after platinum chemotherapy and immunotherapy. AEs occurrence with erdafitinib could be associated with improved mOS and mPFS. Further investigation is needed.

**Acknowledgments:**

E. Gusmaroli, S. Rota, A. Rametta, and P. Giannatempo (Department of Medical Oncology, Fondazione IRCCS Istituto Nazionale dei Tumori, Milan, Italy); R. Miceli (Biostatistics for clinical research unit, Fondazione IRCCS Istituto Nazionale dei Tumori, Milan, Italy); F. Cazzaniga (Oncology Unit, Vimercate Hospital, ASST della Brianza, Vimercate, Italy); N. Cavasin (Department of Medical Oncology, AULSS 2 Marca Trevigiana, Ca’ Foncello Hospital, Treviso, Italy); L. Isella (Medical Oncology Division, ASST Sette Laghi, Varese, Italy); S. Pipitone (Division of Oncology, Department of Oncology and Hematology, University Hospital of Modena, Modena, Italy); M. Fanelli (University Hospital of Udine, Department of Oncology, Udine, Italy).

**Table 1:** Survival outcomes based on the occurrence of AEs.

	mOS months (95% CI)	mPFS months (95% CI)
**Hyperphosphatemia**		
**Yes**	14.1 (8.5, NR)	12.5 (5.3, NR)
**No**	12.5 (5.2, NR)	5.5 (4.1, 13.8)
**Ocular AEs**		
**Yes**	14.1 (7.8, NR)	7.8 (4.4, NR)
**No**	9.7 (5.8, NR)	5.5 (3.3, NR)
**Nail AEs**		
**Yes**	16.0 (10.4, NR)	7.8 (5.5, NR)
**No**	8.5 (4.4, NR)	4.4 (2.2, NR)

